# The College Election Puzzle

**Published:** 1920-11-06

**Authors:** 


### THE MATRONS' AND SISTERS' DEPARTMENT.

### THE COLLEGE ELECTION PUZZLE.

The College of Nursing is greatly exercised about the problem of getting its 19,000 members to elect their Council on representative principles. In this month's number of the *Bulletin* the authorities indulge in a little grumble with their members who cannot be induced to take any interest in the elections; to be frank, until some way can be found of improving the method of election we do not think they ever will do so.

When the time comes for the General Nursing Council to be elected solely by registered nurses the same difficulty is likely to occur. It is one which meets not only nurses in their efforts to govern their own profession, but every borough in the kingdom, nay the High Court of Parliament itself. When nurses have solved their own problem they will have the satisfaction of knowing that they have helped to solve that of many other elective bodies.

We are far from being in a position to attempt a solution of the problem experienced by the College. But it may be useful to indicate where the difficulty lies. The electing members are scattered over the whole kingdom. They belong to very different sections of the nursing world. What rallying-point can serve to unite the territorial divisions of nurses and the different sections of workers?

At present the only members of the College to command anything like general suffrage are certain members of the College who have done it such good service that their names are universally acclaimed, and certain doctors whose names are familiar all over England. One may surmise that these receive far more votes than are needed to secure their admission to the Council. Most nurses, despairing of making any impression by their votes, and ignoring most of the nominees because their names are unknown to them, either do not return the voting paper at all or fill it in quite perfunctorily.

As regards the territorial rights of nurses there can be no doubt that a Council elected mainly from London is not in so strong a position as one in which other parts of the kingdom are represented. At the same time, nothing could well be weaker than a Council elected on the principle of one nurse from each of the local centres, let us say. Probably for the purposes of securing representation the centres would have to be grouped, and if that could be done it, might be possible to lay down the principle that a certain number of Councillors should be voted for in each of three or four divisions. It is only carrying the principle of devolution, which already extends to Scotland and Ireland, a little farther. We do not feel sure that in view of the greater publicity which attaches to the reputation of members of the medical profession, and the pull so to speak which this gives them in an election, that the College will not have to assign the Council a fixed number of medical members which may not be exceeded.

At present hospital or institution nursing is well represented. The problem of securing adequate representation for the two other influential sections of the nursing profession, private nurses and public health (including district) nurses, is a very thorny one. No one can seriously contend that either branch of nursing is "represented" because one or two private and public health nurses are found on the Council. Something more like direct choice on the part of the united members of each section would be necessary to provide representation. What is wanted is a programme, and to prepare a programme implies good organisation and unity of purpose.

Private nurses are at a very grave disadvantage in making their voice heard in the Council chamber. They seldom know more than a small group of comrades out of the large body of electors. They seldom come before the public. Their names do not come into the papers nor have they as a rule the least desire for notoriety. How then is any individual private nurse to secure sufficient votes to ensure her being elected on the Council? Last year the London members united and procured the election of a private nurse whom they knew and had adopted as their candidate. This was so far good, but it did not go nearly far enough.

Public health nurses do not live quite so much in the shade as private nurses. But they are even more cut off from each other. When they rise to positions of influence and become superintendents their names are known throughout a wide district. But then district nurses would not be satisfied to be represented entirely by superintendents. A way must be found through which both private and district nurses may influence the event. That is the crying need at the elections.

